# Indoor Scene Recognition via Object Detection and TF-IDF

**DOI:** 10.3390/jimaging8080209

**Published:** 2022-07-26

**Authors:** Edvard Heikel, Leonardo Espinosa-Leal

**Affiliations:** Department of Business Management and Analytics, Arcada University of Applied Sciences, 00550 Helsinki, Finland; edvardheikel@gmail.com

**Keywords:** scene recognition, object detection, scene classification, TF-IDF

## Abstract

Indoor scene recognition and semantic information can be helpful for social robots. Recently, in the field of indoor scene recognition, researchers have incorporated object-level information and shown improved performances. This paper demonstrates that scene recognition can be performed solely using object-level information in line with these advances. A state-of-the-art object detection model was trained to detect objects typically found in indoor environments and then used to detect objects in scene data. These predicted objects were then used as features to predict room categories. This paper successfully combines approaches conventionally used in computer vision and natural language processing (YOLO and TF-IDF, respectively). These approaches could be further helpful in the field of embodied research and dynamic scene classification, which we elaborate on.

## 1. Introduction

Humans are highly efficient when it comes to contextualizing environments. We can infer information regarding a scene based on observations and extensive prior knowledge we build through experience. This experience can be based on long-term associations we learn through our lifetimes or short-term observations and knowledge that contextualizes current situations. For example, suppose an individual is exploring a campus and walks into a room with several chairs arranged circularly with a large central table. In that case, we might infer that this room is a “seminar room”. Encountering a similar room but in another environment (e.g., an “office space”), we might assign a different label (e.g., “conference room”).

When it comes to designing social robots, it has been argued and shown that semantic level information is essential for indoor scene recognition and navigation [1,2,3,4,5]. If social robots are to be deployed indoors (and, potentially, in other contexts), some level of semantic knowledge must be incorporated. In many simple cases (e.g., a cleaning robot), this might not be necessary. However, if the agent (robot) is to perform “higher” level functions such as target-driven navigation and scene inference, the incorporation of semantic-level information is effective [1,6,7]. Intuitively, if the agent is to locate a particular target object, identifying what room the agent is in and what peripheral (non-target) objects are observed in that room would aid its navigation. Incorporating object-room and object-object semantic-level associations (in combination with computer vision approaches – such as semantic segmentation or object detection) could allow the agent to locate target objects more rapidly [6,8]. Additionally, an agent could incorporate observed objects into its working memory and keep track of contexts and object locations. This would be further useful in navigating an environment, as the agent would often have a sub-par view of its surroundings at any point in time (e.g., it might be staring at the corner of a couch or a blank wall).

Further, when it comes to indoor scene recognition, one could argue that the room category largely depends on the function allocated to said room. For example, consider an empty abode with a certain number of rooms. Some rooms will have predefined determinations of function (such as kitchen, bathroom, garage, among others). However, some rooms are defined by the objects they contain. Moreover, the objects found in these rooms will often be determined by the function assigned to them. For example, a room might be a “home office”, a “bedroom”, or something else. Prior to allocating a function to the room, it could be anything. However, once assigned a function, the objects in the said room would reflect this.

Additionally, a room might be assigned multiple functions, such as a sleeping area and working area (e.g., a bedroom/home office). In which case, the objects observed would likely correspond to objects typically found in both room classes. In this context, object-level representation would be beneficial in disambiguating indoor scenes, as overlapping labels would be appropriate.

To define semantic-level associations, one needs to identify representative data that can be used to quantify these associations. One approach would be to observe how often objects appear together and in what contexts. In the field of natural language processing (NLP), the term frequency-inverse document frequency (TF-IDF) is often used to determine the relevance of words to documents [9,10]. TF-IDF is often used in text classification to identify words typically associated with a specific class of documents [11]. In the context of scene recognition, a similar approach could be utilized to identify what objects (analogous to words) are relevant to rooms (analogous to documents). Particular objects would contain more semantic information relative to other objects. For example, a “chair” is likely to be found in most rooms, whereas a “bed” is most likely found in a bedroom. Additionally, while some objects might be less informative by themselves (e.g., a mirror), they could add semantic information when combined with another object (e.g., mirror-sink). Object co-occurrence has been shown to improve classification models when performing object-level scene classification [12]. Other NLP approaches have also been used to facilitate scene recognition, such as word embeddings [13].

This paper aims to illustrate the benefit of an NLP approach to scene recognition as a proof of concept. Treating scenes as analogous to language would allow one to apply approaches used in NLP to perform and facilitate tasks required by a social robot. This work trained two object detection models to detect objects typically found in indoor environments and applied TF-IDF transformation to classify indoor scenes based on detected objects using a simple machine learning approach. This approach was also implemented using a pre-trained semantic segmentation model.

**Figure 1:**
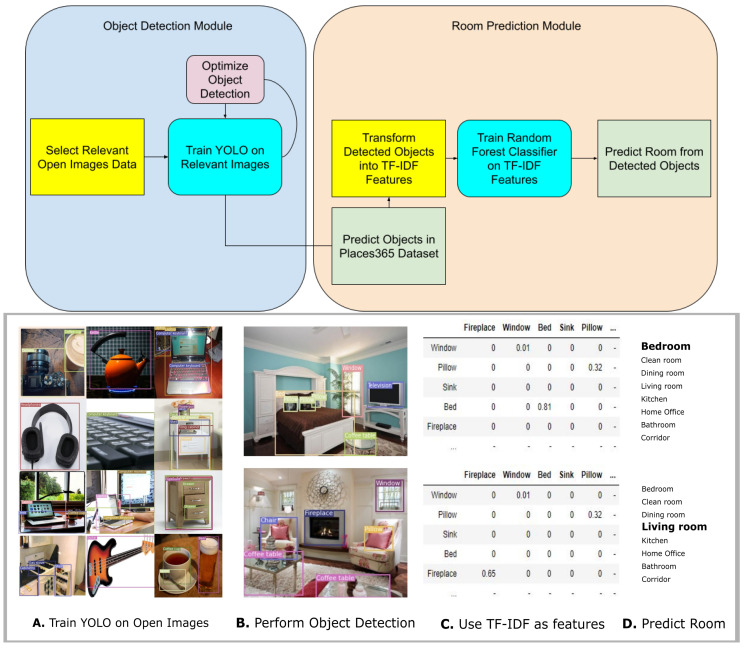
Visualization of the pipeline. *top*: general diagram of the modules for object detection and room prediction, and *bottom*: step-by-step scheme (**A**) Train YOLO to detect indoor objects. (**B**) Perform object detection on scene data (examples use IOD155 with conf. thresh = 0.25). The images are from the ADE20k dataset (**C**) Transform predicted object labels into TF-IDF input features. (**D**) Train classifier to predict room category based on these input features.

This paper is structured as follows; we describe the related research in scene recognition in the scientific literature. Followed by details of the used methodology elaborating on the datasets, the object detection approach, how the objects were transformed into feature inputs, and scene classification (see Figure 1 for an illustration of the pipeline used). Subsequently, the results are reported, followed by a discussion that considers the results and the general approach in the context of wider fields of research.

## 2. Related Work

### Indoor Scene Classification

Traditional methods used for outdoor scene classification do not perform as well when applied to indoor scene classification [14] (see ref. [15] and references therein for a review on scene recognition). This is because indoor scenes tend to have lower variability in global spatial features when compared to outdoor scenes. Early attempts at improving indoor scene classification sought to leverage both local *and* global spatial features by incorporating techniques such as bag-of-visual words [16]. It has been argued that indoor scene classification has two main challenges, (1) low inter-class variance between scene categories and (2) ambiguity regarding scene labeling [17]. This ambiguity could potentially arise due to an overlap between room functionality and labels associated with particular rooms. For example, if a room has a dining table, one couch, and one television, is it a dining room or a living room? How would one demarcate areas in a studio flat with an attached kitchen? Does a counter separate the kitchen or not? The overlap of functionality in room categories is a challenge to be considered.

More recently, techniques have focused on scene classification by leveraging object-level semantic information [18,19,20,21]. In 2019, Chen and colleagues [13] investigated scene classification by combining traditional scene classification techniques with NLP methods. Using a convolutional neural network (CNN) module, they generated an ordered top-5 prediction for a given image and segmented the scene using a scene parser module. These segmented objects were then passed through a word embedding module that refined the top-5 predictions and improved indoor scene classification performances. They trained and tested their model within three super-categories: school, shopping mall, and home. They reasoned that while GPS tracking would be sufficient in determining the general setting of a potential agent, it would not be sufficient in determining the exact location and room that the agent would find itself in. Considering overlap between many scene categories, refining the potential room classes to setting specific choices could reduce the limitations of low scene variability. For example, by using GPS, one could determine that the agent is on a school campus and thereby use an indoor room classification model trained on indoor school settings to predict the room category that the agent is in.

In addition to methodological limitations in finding the best algorithms for indoor scene recognition, it is necessary to address the technical elements of implementing these. The method demonstrated here builds on previous work by providing a relatively simplistic approach that leverages the speed of a widely used object detection network: YOLO (You Only Look Once) [22] and a simple NLP approach that is not computationally demanding. In the context of robotics, where incoming visual data relies on frames, having approaches that can process information close to the speed of incoming frame rates could be a boon. There are papers that demonstrate the speed of YOLO [22] and while improved accuracy is always desirable-being able to perform the same task at less computational cost should also be valued.

Teder and colleagues [12] investigated various word embedding approaches (Latent semantic analysis [23] and word2vec) and whether object-level distributions and co-occurrences contributed meaningful semantic information to scene recognition. They compared Residual networks [24] and VGG networks [25] and how well they performed when fusing word embeddings in the final CNN layer. They observed improved scene recognition performances when incorporating object-level semantic information.

Our approach is closely related to both methods [12,13]. While both incorporate object-level semantic information in scene recognition, our approach performs scene recognition solely through object-level representations. This means that we represent a scene not through the embedded objects but by means of vector representations of these objects.

Zhou et al. [26] have recently implemented a Bayesian approach for scene recognition. The authors implemented an improved object model (IOM) enriched from a Bayesian perspective (BIOM) to find object co-occurrences and pairwise object relations. These models are incorporated into a pretrained CNN model (placesCNN) and tested on the reduced places365 dataset (n = 7 and 14 classes) and SUN RGB-D dataset [27]. PlacesCNN can be seen as the original baseline for scene recognition on the places365 dataset [28]. In the same direction, Miao and colleagues [29] propose an Object-to-Scene (OTS) method, which extracts object features and learns object relations to recognize indoor scenes. More recently, Labinghisa et al. [30] proposed a method called image-based indoor location awareness algorithm (IILAA) in combination with a clustering algorithm, with state-of-the-art performance on the MIT67 dataset [14].

## 3. Methodology

### 3.1. Datasets

Open Images V6 [31] was used to train YOLOv5 [32] to perform object detection using 90 classes (Indoor Object Detection 90-IOD90) and 155 classes (IOD155) that one would reasonably expect to find in indoor settings (e.g., oven, dining table, TV, keyboard, bed, flower, sink, laptop, wrench, etc.). For IOD90, 309,762 images were used for training, 6307 were used for validation, and 18,644 for testing. For IOD155, 468,579 images were used for training, 11,717 images were used for validation, and 34,907 for testing. The Open Images dataset contains annotated images, and the images were selected based on whether object classes were present. Irrelevant annotations were ignored, and only target object classes were used. In Figure 1 an overall technical flow of the pipeline proposed in this work is depicted, as well as a step-by-step scheme. Moreover, the pseudocode of the whole algorithm is presented in Algorithm 1.

For scene recognition, eight indoor room classes were selected from the Places365 dataset [28] (bathroom, bedroom, corridor, clean-room, kitchen, home-office, living-room, and dining-room). Five thousand images were selected for training and validation for all categories apart from “clean-room”, with 3871 images. The number of images used for training was 38,871, of which a balanced 20% were used for validation. For testing, 100 images were used for each category (for a total of 800) and correspond to the Places365 designated validation set. The original Places365 testing set does not contain labels as it is part of an ongoing challenge; therefore, a customized validation/testing approach was required.
**Algorithm 1** Pseudocode of the proposed approach.1:**procedure**Requirements:2:    A working object detection model        ▹ either custom or pretrained3:    A dataset D of scene data                                     ▹ with labeled rooms4:**procedure** FOR PERFORMING ROOM CLASSIFICATION5:     **for** *Image* in dataset D **do**6:        DetectObjects(*Image*)7:        TrainValTest.split(D)8:    CountVectorizer()9:    TF-IDF()10:    TrainClassifier()                                    ▹ for predicting room category11:    PredictRooms()                 ▹ evaluate room classification performance

### 3.2. Object Detection Modules

We trained a recent implementation of YOLO [22,33], YOLOv5L (See https://github.com/ultralytics/yolov5, accessed on 13 November 2021), to detect predefined objects using the Open Images V6 dataset. Of the 600 trainable objects in this dataset, 90 and 155 object classes were selected to be used. For the used images, the annotations were converted to a PASCAL VOC format. YOLO was used because it is one of the fastest object detection methods currently available. In the field of embodied agents and reinforcement learning, the improved speed of YOLO could be beneficial when performing studies investigating reinforcement learning and object navigation. This is because reinforcement learning is already computationally expensive, and using a relatively “simple” object detection framework could be beneficial [34,35]. Additionally, we are aware of the current controversy revolving around YOLOv4 [36] and YOLOv5 [32] and have no reason to select one over the other. The current study aims not to optimize the object detection task, but instead to utilize object detection (trained on custom data) in a scene recognition task. We have no opinion regarding the appropriateness of which version to use; we have used the Jocher and colleagues [32] implementation of YOLO because it is compatible with PyTorch [37].

YOLOv5 was trained using the default hyperparameters for 100 epochs in batches of 32. YOLOv5 uses standard Non-Max Suppression (NMS) in post-processing. It provides the option for image augmentation and while creating mosaics in the training phase we did not initialize the image augmentation preprocessing option. YOLOv5 uses anchor boxes and determines them using an “AutoAnchor” [38]. For testing model performance, a confidence threshold of 0.001 was used. The same confidence threshold was used to detect objects for the indoor scene classification and 0.25, 0.50, and 0.75. However, increasing the confidence threshold was negatively associated with indoor scene classification accuracies. This is because as the confidence threshold increases, fewer objects are detected, and so the number of images without detected objects increases. It would be interesting to see how false positives and false negatives affect classification performances in scene recognition. However, as the images used for scene recognition had no object-level annotations, this could not be tested. It could be that if a false positive is consistent enough in a scene category, it could be “informative” and be used in scene classification despite being mislabelled; unfortunately, this was not something we could empirically expand on.

A pre-trained semantic segmentation model (Xception [39]) was also used. The semantic segmentation model contained 150 classes relevant to the ADE20k dataset [40]. ADE20k is a dataset that provides semantic segmentation labels for images containing scene categories corresponding to the Places365 dataset. It is important to note that the 150 classes used in the segmentation model also include classes relevant to outdoor scenes and are not exclusive to objects found in indoor environments.

### 3.3. Object-Level Scene Classification Module

We generated object-level predictions (with object detection and semantic segmentation, separately) that were used as features in relevant images in the Places365 dataset using the Object Detection Modules. These features were then transformed using TF-IDF and Count Vectorizer with the default parameters, where each image was treated as a document. Count Vectorizer is a standard method used in NLP and it is used to convert corpora into a matrix of token counts of words. In this case, it is used to count detected objects in order to identify their frequency across our scene data. It is crucial to keep in mind that Places365 does not have object-level annotations. So, the input features for scene classification represent predicted objects – as opposed to concrete and well-defined labels.

Term frequency (TF—Equation (1)) is defined as the frequency that the term *t* appears in the document *d* and inverse document frequency (IDF) is a metric used to identify how much information the term provides. IDF (Equation (2)) is calculated as the logarithm of the total number of documents *N* divided by the number of documents where the term *t* appears (i.e., in regard to this study: the number of images, where the object is present) and corresponds to how frequent or rare a term is in a collection of documents. Term frequency-inverse document frequency (TF-IDF—Equation (3)) is calculated as the product of TF and IDF. The more relevant a word is to a document, the higher the TF-IDF score (on a scale of 0–1). TF-IDF was calculated using Scikit-Learn [41].

(1) $$tf\left(t,d\right)=\frac{f_{t,d}}{\sum_{t^{\prime }\in d}f_{t^{\prime },d}}$$

(2) $$idf\left(t,D\right)=\log\frac{N}{\left|d\in :t\in d\right|}$$

(3) tfidf(t,d,D)=tf(t,d)×idf(t,D)

The same weighting can be applied to objects and scenes if the term for object and document for an image is substituted. Common objects observed in most scenes would be weighted down, and rare objects would have an increased weighting. For example, one would expect “oven” to be a relatively infrequent object but often observed in kitchen settings. In this context, ovens would have a higher TF-IDF weighting than a more frequent and less informative object (e.g., a chair).

Using a Bag-of-Words (BoW) [42] approach, these features correspond to a vectorization of the frequency of occurrence for objects in the target room categories. This vector space was then used to train a classifier to predict a room category based on observed (predicted) objects. In NLP, BoW approaches can be limited because they do not capture the structural sequence and order of words so other approaches might be more appropriate. However, in static scene recognition, there is no sequential order (which could be more relevant in dynamic scene processing), so this limitation is less applicable here.

A random forest classifier was used to predict scene category from observed (predicted) objects in images using Scikit-Learn [41] with *n* = 1000 estimators, the minimum number of samples is 2, and an unlimited number of leaf nodes. This was applied to all object detection methods (IOD90 and IOD155) and semantic segmentation (Xception). Other machine learning models were tested (linear regression and support vector machine) however random forest was superior in all cases and gave the best results.

## 4. Results

### 4.1. Evaluating Object Detection

Using a confidence threshold of 0.001, object detection for the 90 indoor classes had a precision of 0.526, recall of 0.601, mean average precision at 0.5 IOU (mAP@0.5) of 0.553 and a mAP@0.5:0.95 (mean average precision at IOUs from 0.50 to 0.95 at 0.05 increments) of 0.416. Object detection for 155 indoor classes had a precision of 0.455, recall of 0.469, mAP@0.5 of 0.417 and a mAP@0.5:0.95 of 0.309. These results are summarized in Table 1. and correspond to 100 epochs of training using default hyperparameters and evaluated on the Open Images testing data.

### 4.2. Scene Classification with IOD90

**Figure 2:**
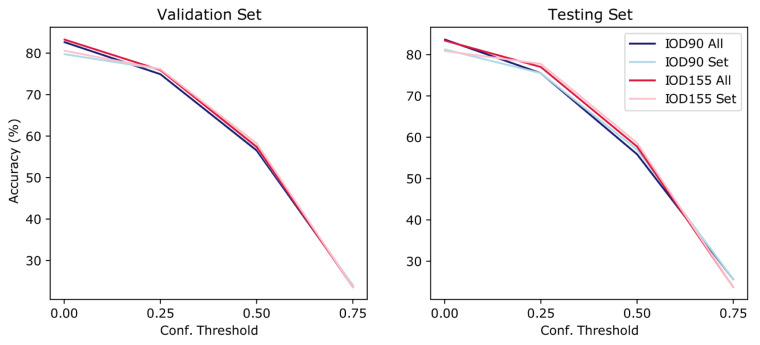
Scene Recognition for IOD90 & IOD155—visualization of results across conf. thresholds (0.001, 0.25, 0.50, 0.75) for validation and testing sets. Also displayed are whether all detected objects or singular instances (sets of objects) are used in predicting room category.

Scene recognition using 90 classes of objects (IOD90) and a confidence threshold of 0.001 achieved an accuracy of 82.53% on the validation set (test: 83.63%) and using a confidence threshold of 0.25 achieved an accuracy of 74.92% (test: 75.50%). Using a confidence threshold of 0.50, an accuracy of 56.65% on the validation set (test: 55.88%) was achieved. Using a highly restrictive confidence threshold of 0.75, the accuracy was close to chance at 23.97% (test: 25.63%). Increased confidence thresholds were associated with lower accuracy scores (see Figure 2 for a visual summary).

The number of detected objects might explain this. Using a confidence threshold of 0.001, a mean of 299.02 (std: 6.00, range: 12–300) number of objects per image were detected and using a confidence threshold of 0.25, a mean of 44.69 (std: 39.28, range: 0–300) objects per image was detected. An average of 10.05 (std: 13.30, range: 0–277) and 0.74 (std: 2.35, range: 0–91) number of objects were detected using confidence threshold of 0.50 and 0.75, respectively. Further, increasing thresholds resulted in more images having no predicted objects, which influenced the performance of the object-level classification of scenes. For example, with a 0.001 confidence threshold, all 90 classes were detected, with all images having at least one detected object (i.e., 100%). With a 0.25 confidence threshold, 89 object classes were detected, with 92.07% of images having at least one detected object. Using a confidence threshold of 0.50, 80 classes were detected across 66.53% of all images and using a 0.75 confidence threshold, 55 objects were detected across 15.21% of images.

Considering how many objects were detected on average for a confidence threshold of 0.001, scene classification was tested using the only single occurrence of objects detected (i.e., using only the presence of an object class, as opposed to all detected instances of objects). When ignoring duplicate objects, IOD90 achieved a 79.74% accuracy (test: 81.25%) with a 0.001 confidence threshold on the validation set and an accuracy of 76.10% (test: 75.50%) using 0.25. An accuracy of 57.21% was observed on the validation set (test: 57.00%) using a threshold of 0.50, and an accuracy of 23.99% was observed (test: 25.63%) using a threshold of 0.75. Only slight changes in accuracy were observed: −2.79%, +1.18%, +0.56% and +0.02% for confidence thresholds of 0.001, 0.25, 0.50 and 0.75, respectively, on the validation sets when using sets of objects.

### 4.3. Scene Classification with IOD155

Using a model trained to detect 155 object classes (IOD155) an accuracy of 83.25% on the validation set (test: 83.38%) was observed using a confidence threshold of 0.001 and an accuracy of 75.91% (test: 77.00%) was observed using a threshold of 0.25. A threshold of 0.50 achieved an accuracy of 57.43% on the validation set (test: 57.75%) and a threshold of 0.75 achieved an accuracy of 23.61% (test: 23.75%).

Again, this could be explained by the number of detected objects. On average, 298.98 (std: 11.48, range: 12–300), 48.57 (std: 43.37, range: 0–300), 10.81 (std: 14.47, range: 0–300), 0.80 (std: 2.68, range: 0–162) objects were detected per image using confidence thresholds of 0.001, 0.25, 0.50, 0.72—respectively. Out of 155 classes, 135 objects were detected at least once using a threshold of 0.001 across all (100%) images, and 125 objects were detected at least once using a threshold of 0.25 across 90% of the images. Using a threshold of 0.50, 106 object classes were detected across 66.89% of the images, while 60 object classes were detected in 15.91% of the images at least once using a threshold of 0.75.

When ignoring duplicate objects detected, a confidence threshold of 0.001 achieved 80.57% accuracy (test: 80.88%), a threshold of 0.25 achieved an accuracy of 76.14% (test: 77.75%), a threshold of 0.50 achieved an accuracy of 58.25% (test: 58.63%) and 0.75 achieved an accuracy of 23.64% (test: 23.75%). Again, only slight differences in accuracy were observed: −2.68%, +0.23%, +0.82%, +0.03% for confidence thresholds of 0.001, 0.25, 0.50 and 0.75, respectively, on the validation set.

### 4.4. Scene Classification with Semantic Segmentation

Using a pre-trained semantic segmentation model, an accuracy of 80% was achieved on the validation set and 71.41% accuracy on the testing set. Across all data, semantic segmentation detected 149 object classes. Unlike YOLO, semantic segmentation uses masking, and the implementation here does not allow for the counting of objects detected (apart from the number of classes). On average, 16.37 (std: 6.50, range: 1–47) object classes were detected per image. The most commonly segmented classes were “wall”, “floor”, and “ceiling”, which is to be expected for indoor environments. However, due to their relatively high-frequency and shared commonality across room categories, TF-IDF should down weight their importance. All images contained at least one instance of those classes; when ignoring those semantic labels, at least one object was detected in 99.91% of all images.

### 4.5. Model Comparisons

Identifying relevant benchmarks for this study is no trivial task. Table 2 compares a selection of scene recognition models. Often when looking at a large number of classes for scene data (e.g., the full ADE20k and Places365 datasets), researchers compare model performances regarding their accuracy for the Top-1 prediction and the Top-5 predictions. This is due to overlap and ambiguity; however, as this study only has eight room categories, a Top-5 accuracy would not be appropriate. Furthermore, the room categories were confined to rooms typically found in residential homes. Chen and colleagues [13] use the Top-5 predictions and refine their predictions based on word embeddings. Our results are comparable to theirs as they use the same dataset with more room categories represented in their “home” data (*n* = 14). However, they select their testing set from the Places365 training set and use the Places 365 testing set as their validation set, which differs from this study. Newer approaches where the semantic relationships among the objects [26] and transfer object learning [29] are included for scene recognition improve the state-of-the-art by around 2.0%.

Despite the differences in the number of room categories, the performances on the testing set for this study more closely resemble their validation set (both in terms of results and likely overlapping scene data).

### 4.6. Experimental Settings

The models were implemented in the Pytorch library and trained using an NVIDIA Volta V100 GPU with 10 cores from a Xeon Gold 6230 processor, with 32Gb of RAM. The parameters used during the training stage were a batch size set to 32 and 100 epochs. The Adam optimization algorithm with a base learning rate of 0.1 for minimization, while momentum and weight decay are set to 1.0 and 1 ×$10^{-5}$, respectively. The obtained models and datasets (IOD155 and IOD90) are available for reproducibility (see Reference [43]).

## 5. Discussion

This study aimed to demonstrate the benefit of NLP approaches to scene recognition and further illustrate object-level importance. As demonstrated, indoor scene classification can be performed solely from object-level information by combining TF-IDF weighting with detected objects in static scenes.

Researchers often use semantic information to facilitate high-level functions such as object-goal-directed navigation and exploration in embodied research. The object detection models developed here could further advance this field, as they often rely on pre-trained models that contain a limited number of classes relevant to indoor scenes, such as MS-COCO [6]. YOLO was trained to perform object detection on 90 and 155 object classes relevant to indoor scenes, which could be beneficial to the field.

When evaluating the object detection models, IOD90 performed better than IOD155; however, IOD155 performed better in scene classification. This illustrates one of the limitations of this study. Object-level annotations for the scene data (Places365) were not available, so evaluating the object-level prediction on the scene data is not possible. Generally, lower confidence thresholds were associated with improved scene recognition, which is likely due to more input features for the classification task.

Both IOD90 and IOD155 performed better than the semantic segmentation model when used as input features for classification. This is likely due to IOD90 and IOD155 incorporating more semantic labels specific to indoor scenes. The semantic segmentation model also contained semantic labels associated with all other scene categories from the Places365 dataset, including outdoor scenes. Therefore, one cannot infer that one approach is better than the other. However, in this case, it is likely that object detection performed better due to having more relevant indoor object classes.

Interestingly, only slight deviations in classification performance were observed when using instances of objects detected (i.e., sets of objects). In the context of dynamic scene classification, this might be an interesting line of future research. When exploring a room, a proportion of the relevant frames could include a suboptimal field of view. Recently encountered objects could be stored in working memory to allow for contextualizing the immediate scene/frame. However, performing object detection over all frames could lead to large collections of “encountered” objects - despite the actual number of objects being far lower (due to the same object/s being present across frames). Using sets of recently encountered objects could simplify this task.

## 6. Conclusions

This paper illustrates the relevance of objects and NLP approaches to indoor scene classification. These models were then used to predict objects in unlabelled scene data by training YOLO to detect indoor objects. These predicted objects were then used to train a classifier, using object TF-IDF values as input features to classify room categories.

This approach could yield further benefits to static and dynamic indoor scene classification and could also be beneficial for embodied research. Given the presented approach’s simplicity, the proposed implementation can be deployed easily on low-cost hardware, which is the case with most commercially available humanoid robots. Some limitations include the lack of semantic relationships among the objects in rooms once these are detected, as well as the absence of learning about the room’s composition once the classification is performed. This last limitation is essential in case of dynamic changes if the various aspects of the room change, so there is no need to run the algorithm every time in the same location. Future research directions include addressing these limitations, and, more importantly, our goal is to deploy the proposed algorithm in assistive robots, particularly for elderly care.

## Figures and Tables

**Table 1:** Evaluating YOLO.

Model	Precision	Recall	mAP@.50	mAP@.50:.95
IOD90	0.526	0.601	0.553	0.416
IOD155	0.455	0.469	0.417	0.309

**Table 2:** Scene Recognition—Model Comparisons (IOD90 & IOD155 conf. thresh = 0.001).

Dataset		Top-1		Top-5	
		Val	Test	Val	Test
ADE20K					
	ResNet18+LSA [12]	53.77%	-	75.65%	-
Places365					
	VGG [28]	55.24%	55.19%	84.91%	85.01%
	ResNet152 [28]	53.63%	54.65%	85.08%	85.07%
Places365-Home					
	ResNet50 [13]	83.46%	92.03%	-	-
	ResNet50+Word2Vec [13]	83.67%	93.27%	-	-
	CBORM [26]	85.80%	-	-	-
	OTS [29]	85.90%	-	-	-
**this work**	**IOD155 + tfidf**	**83.25%**	**83.38%**	-	-
	**IOD90 + tfidf**	**82.53%**	**83.63%**	-	-
	**Xception + tfidf**	**80.00%**	**71.41%**	-	-

## Data Availability

Open Images V6 is available at https://storage.googleapis.com/openimages/web/index.html, (accessed on 1 July 2021). Places365 is available at http://places2.csail.mit.edu/download.html, (accessed on 1 July 2021). Models and data are available at https://doi.org/10.5281/zenodo.6792296, (accessed on 1 July 2021).
